# The immunoregulatory effect of short-chain fatty acids in type 2 diabetes mellitus

**DOI:** 10.3389/fnut.2026.1807302

**Published:** 2026-04-10

**Authors:** Jiaxin Li, Zhongmin Lv, Qi Fan, Peinan Fu, Ning Zhou, Jiefang Deng, Huimin Qi, Meng Yang

**Affiliations:** 1 Jiangsu Food and Pharmaceutical Science College, Huai’an, Jiangsu, China; 2 Asymchem Life Science (Tianjin) Co., Ltd., Tianjin, China; 3 Heilongjiang University of Chinese Medicine, Haerbin, Heilongjiang, China

**Keywords:** SCFAs, T2DM, GPCR, HDAC, innate immunity, adaptive immunity

## Abstract

Short-chain fatty acids (SCFAs) are the primary metabolites of dietary fiber fermented by intestinal flora. They play a systemic role in the immune regulation of type 2 diabetes mellitus (T2DM) by integrating receptor-mediated signaling and epigenetic regulatory mechanisms. At the receptor pathway level, SCFAs activate G protein-coupled receptors such as GPR41/43/109 A, initiate downstream signaling cascades including MAPK, NF-κB, and mTOR/STAT3, and thereby achieve rapid modulation of immune cell function; at the epigenetic regulatory level, SCFAs induce chromatin remodeling and gene expression reprogramming by inhibiting histone deacetylase (HDAC) activity, giving immune cells long-term functional memory. These two pathways act coordinately to broadly regulate the functional status of innate and adaptive immune cells. In innate immune cells, SCFAs influence macrophage polarization, neutrophil activation, dendritic cell antigen presentation, mast cell degranulation, and eosinophil-mediated immune homeostasis; in adaptive immune cells, SCFAs regulate the differentiation of CD4 + T cell subsets, CD8 + T cell effector function, regulatory T cell stability, B cell antibody production and cytokine secretion of congenital lymphocytes (ILCs). These immunomodulatory effects are integrated in multiple metabolic organs such as adipose tissue, liver, islet and intestine to collectively improve T2DM-related chronic inflammation and insulin resistance. Investigation of SCFAs reveals the molecular basis of the interaction between intestinal flora and host immune metabolism, and provides a theoretical foundation for the prevention and treatment of T2DM based on dietary intervention or microecological regulation.

## 1 Introduction

According to the data of the International Diabetes Federation (IDF) in 2021, there are about 537 million patients with T2DM in the world, and the prevalence rate in China is as high as 10.6% (1). The core pathophysiological manifestations of T2DM are β-cell dysfunction, insulin resistance (IR) and chronic inflammation, which are interconnected and jointly disrupt the maintenance of blood glucose homeostasis (2). Inflammation is an immune response activated by the body to protect itself from pathogens, and it also contributes to tissue repair and recovery. This response shows protective effects when inflammation is mild and limited in duration. However, in recent decades, systemic chronic inflammation has been shown to play an important role in the pathogenesis and progression of major fatal diseases such as cancer, heart disease, diabetes and neurodegenerative diseases (3). Inflammation has a central role in the development and progression of diabetes. Studies have shown that chronic low-grade inflammation is associated with IR, which is a recognized characteristic of T2DM. Inflammation promotes the activation of pro-inflammatory cytokine signaling, thereby blocking the insulin receptor of islet β cells and impairing the body’s effective response to insulin. Inflammation further weakens the cellular transmission of insulin signals, making it increasingly difficult for the body to respond properly to insulin. This outcome is referred to as pancreatic IR and represents an indicator of T2DM (4). Inflammation, oxidative stress and activation of protein kinase C (PKC) are known to reduce insulin receptor substrate 1 (IRS1) activity by increasing serine/threonine phosphorylation and decreasing tyrosine phosphorylation, all of which have been shown to impair insulin signaling (5).

In addition, there is a close association between inflammation and IR, and the advanced glycation end products (AGE)/RAGE/NF-κB axis represents the core of this pathological process (6).

**Figure 1:**
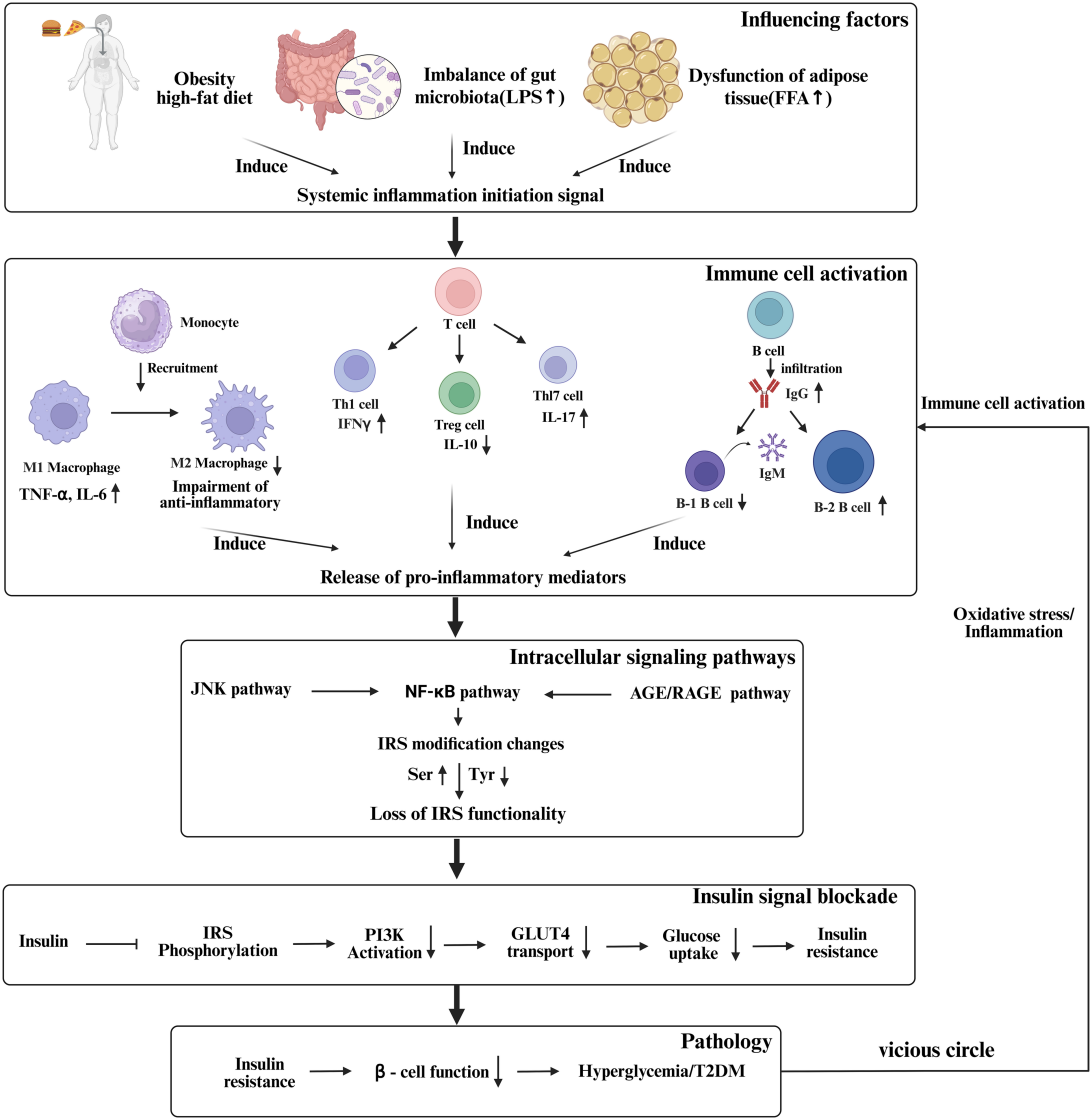
The core mechanism of inflammation, immune system, and T2DM.

High levels of proinflammatory cytokines, such as tumor necrosis factor-α (TNF-α), interleukin (IL)-1β and IL-6, are present in patients with T2DM. These cytokines are strongly associated with insulin resistance and β-cell dysfunction (7). It is noteworthy that obese individuals with only elevated inflammatory markers also show a significantly increased risk of developing T2DM in the future, suggesting that inflammatory processes precede the disease and contribute to the development of IR (8). Inflammatory mediators can also activate C-Jun N-terminal kinase (JNK) and NF-κB to catalyze the phosphorylation of serine residues of IRS protein, rather than tyrosine phosphorylation under physiological conditions. Serine-phosphorylated IRS is unable to effectively recruit downstream PI3K and is even targeted for degradation, thereby blocking the intracellular transmission of insulin signals—a key molecular event in inflammation-induced IR (9). This inflammatory state differs from acute infection, presenting as chronic low-grade inflammation-mild but persistent, and involving systemic alterations across the immune system (7) (Figure 1).

The function of the immune system is based on the proliferation and differentiation of immune cells and various types of cytokines. Immune cells require metabolic support to provide energy and substrates, such as glucose, amino acids, phospholipids and fatty acids; therefore, the immune system also regulates metabolism to ensure adequate energy supply (10). Cytokines in immune metabolism not only participate in immune regulation, but also act as mediators of energy metabolism. Some metabolites, such as glucose, in addition to providing energy, can also function as signaling mediators that activate responses to pathogens. In general, immunity plays a vital role in metabolic homeostasis, and immune imbalance may lead to metabolic diseases including T2DM. The occurrence of T2DM corresponds with changes in the immune cell spectrum, including alterations in the mononuclear macrophage system, B lymphocytes, T lymphocytes, natural killer (NK) cells and innate lymphocytes (ILCs). In general, the immune cell profile is characterized by pro-inflammatory proliferation, differentiation and phenotypes, including increased M1-like macrophages, helper T (Th) 1 cells, Th17 cells, CD8 + cells, and antibody-producing B-2 cells (11–13), and down-regulation of M2-like macrophages, Th2 cells, regulatory T cells (Tregs), IgM-producing B-1 cells and ILCs subsets (such as ILC2 and ILC3) (14). This immune cell–cell interaction triggers pathogenic inflammation by releasing a series of pro-inflammatory cytokines (such as TNF-α, TNF-β, IL-1β, IL-2, IL-6, IL-17, interferon (IFN) -α, IFN-γ) and IgG in circulating and glucose-metabolizing tissues such as adipose tissue, liver, muscle and pancreas, further disturbing metabolic balance and eventually leading to islet β-cell dysfunction, glucose intolerance and IR (15).

Gut microbiota is a complex microecosystem, and maintaining a mutually beneficial symbiotic relationship with human hosts is essential for health. A typical manifestation of this symbiotic interaction is that bacteria ferment dietary fiber to produce SCFAs, while the host provides indigestible carbohydrates as growth substrates; the bacteria subsequently generate SCFAs that feed back to the host, providing energy for colon cells, reducing inflammation and regulating satiety. Intestinal microecological imbalance may lead to various chronic diseases, among which insufficient SCFAs production is closely associated with T2DM (16). Increasing evidence indicates that SCFAs play a key role in shaping both the local and peripheral immune system, which subsequently affects host metabolism through inflammatory pathways. The function of the immune system depends on the proliferation and differentiation of immune cells. This process requires metabolic support to provide energy and substrates (17); cytokines in immune cells are not only involved in immune regulation but also function as mediators of energy metabolism, and some metabolites (such as glucose) can also act as signaling molecules that activate immune responses (17). As the primary products of dietary fiber fermentation by intestinal flora, acetate, propionate and butyrate represent the three main SCFAs. They are not only energy sources for intestinal epithelial cells, but also systemically regulate the function and fate of immune cells through multiple mechanisms, thereby influencing the insulin sensitivity of distal metabolic organs and functioning as the “molecular language” of microbial signals and host immune communication.

**Figure 2:**
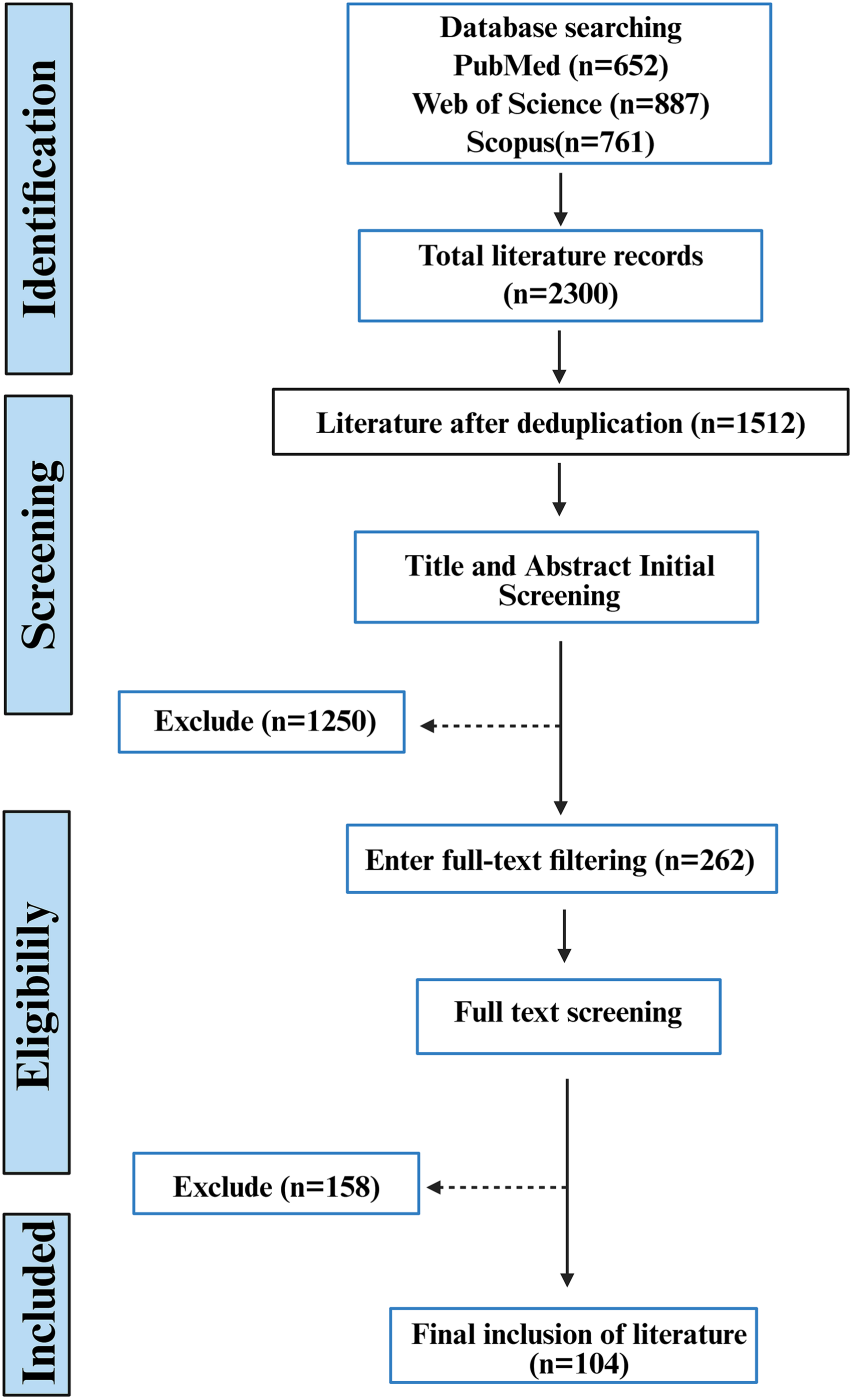
Literature screening flow chart.

However, the existing mechanistic research remains fragmented. Although the protective effect of SCFAs in T2DM has been widely recognized, many studies focus on only specific aspects of their mechanisms: some emphasize receptor-mediated signaling pathways, others highlight epigenetic regulation, and some examine their direct regulation of immune cell metabolism. Although these three mechanisms coexist within the same biological process, they are frequently discussed independently and lack an integrated perspective. In reality, there is a spatiotemporal synergy and molecular dialogue between the rapid transduction of receptor signals and the long-term programming of epigenetic regulation, which together determine the direction of functional remodeling of immune cells. Therefore, understanding the functioning of the various components of the immune system is crucial for clarifying the inflammatory process in T2DM. Based on this background, this paper aims to construct an integrated mechanistic framework from molecular recognition to systemic effects by organizing relevant literature from recent years, and systematically explains how SCFAs achieve immune-metabolic homeostasis regulation in T2DM through a complete chain of receptor signal initiation, epigenetic programming, immune cell function remodeling, and metabolic improvement execution (Figure 2).

## 2 The potential inflammatory pathways of SCFAs in T2DM

**Figure 3:**
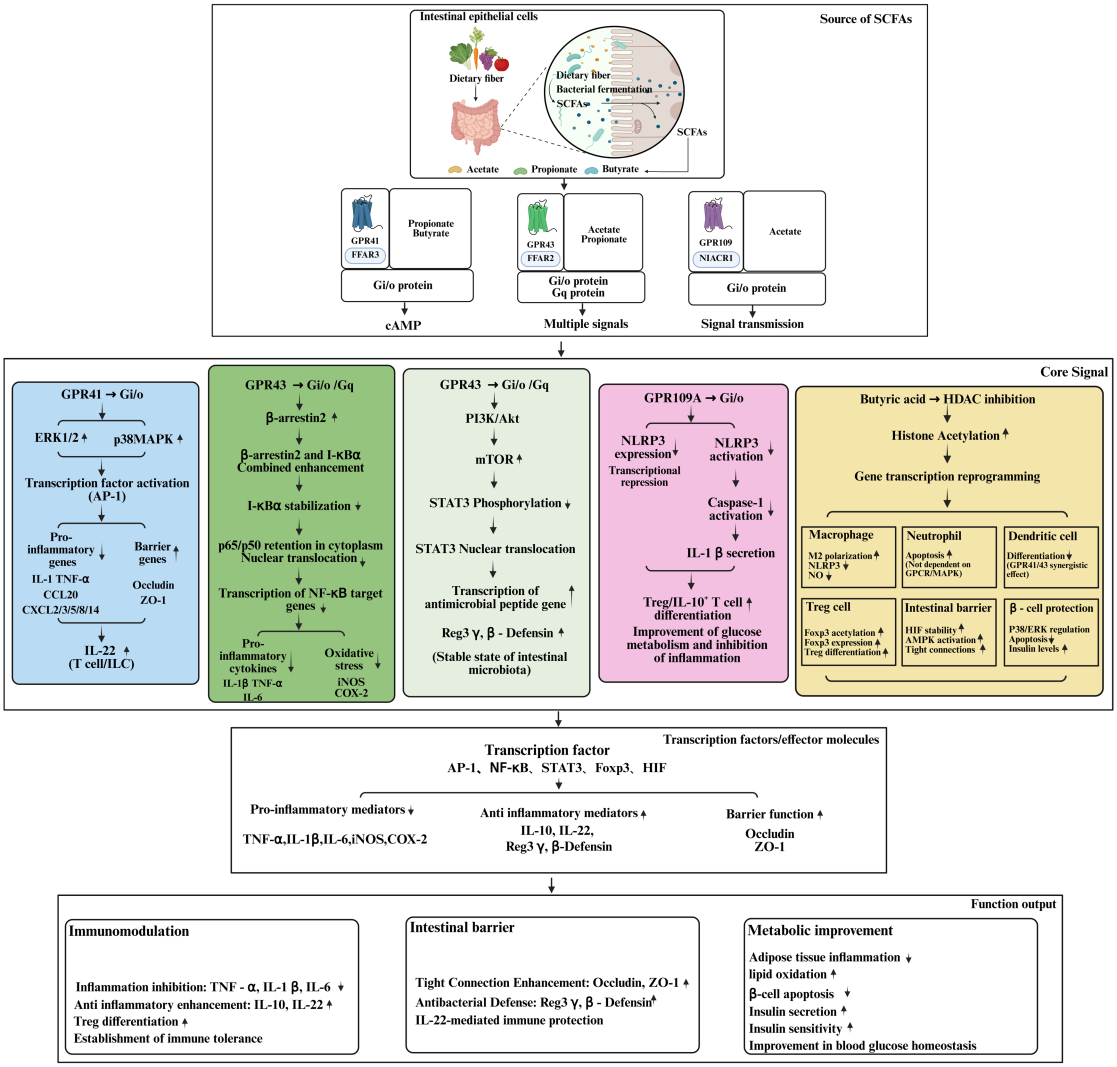
Signaling pathway diagram: the core signaling network of SCFAs regulating inflammation and immunity.

SCFAs are fatty acids with fewer than 6 carbon atoms and short aliphatic carbon chains (18). SCFAs include formic acid, acetate, propionate, butyrate and valeric acid. Due to their relatively short hydrophobic chain and hydrophilic carboxyl group, short-chain fatty acids exhibit high water solubility and can be efficiently absorbed or transported by cells (18). SCFAs are produced through fermentation under the catalytic activity of colonic microbiota. Under anaerobic conditions, bacteria partially decompose sugar molecules during this process. Carbohydrates are an important source of SCFAs, but other dietary components such as proteins and peptides can also generate small amounts of SCFAs (19). However, host digestive enzymes can efficiently degrade these SCFAs precursors in the upper gastrointestinal tract, preventing them from reaching the colon and thereby limiting their utilization by microbiota for SCFAs production. On the other hand, indigestible oligosaccharides and fibers, such as fructooligosaccharides, inulin, pectin and arabinoxylans, serve as excellent sources of SCFAs. However, insoluble fibres such as chitin and cellulose are not easily degraded by the microbiome and therefore do not produce substantial amounts of SCFAs. Although no definitive conclusions have been reached through extensive bacterial isolation and metagenomic investigations, current evidence indicates that different bacterial species show substantial variation in the genetic composition of enzymes involved in SCFAs synthesis (Figure 3) (20).

### 2.1 SCFAs receptor pathway

Activation of free fatty acid receptor 2 and 3 (FFAR2/3) is one of the principal mechanisms through which SCFAs influence target cells (21, 22). The human body produces SCFAs through microbial fermentation processes and utilizes them as energy substrates in multiple biological activities (23). SCFAs are generated by microbial fermentation of indigestible dietary fibers in the intestine. In contrast, long-chain and medium-chain free fatty acids are mainly derived from dietary triglycerides (24). In the past, free fatty acids were regarded primarily as energy sources. It is now recognized that they bind to a class of cell surface receptors known as free fatty acid receptors (FFARs), which trigger intracellular signal transduction and subsequently produce a range of cellular and tissue responses. All FFARs belong to the G protein-coupled receptor (GPCR) family and are essential for regulating metabolism, immune function, inflammatory processes, and the secretion of hormones and neurotransmitters (24). There are currently four distinct types of FFARs; FFAR1 (formerly known as GPR40) and FFAR4 (formerly known as GPR120) mediate the action of long-chain and medium-chain free fatty acids. In contrast, FFAR3 (previously referred to as GPR41) and FFAR2 (previously referred to as GPR43) function primarily as short-chain fatty acid receptors (SCFARs) that respond to SCFAs such as butyrate, acetate and propionate (25). Since FFAR2/3 are widely expressed in the gastrointestinal tract and other tissues, they participate in numerous physiological and cellular processes. The heart, adipose tissue, spleen, liver, bone marrow, lung, pancreas, brain, colon and muscle express these two receptors in different patterns (26). A variety of human diseases, including cardiovascular diseases (CVDs), colitis, inflammatory bowel disease (IBD), T2DM, and obesity, are influenced by the widespread expression of FFAR2/3 molecules (26). When FFAR2 or FFAR3 binds endogenous SCFAs on the cell surface, heterotrimeric G proteins are activated within the cell. FFAR2 and FFAR3 are G protein-coupled receptors characterized by a common seven-transmembrane structural motif. Some GPCRs, such as GPR41, GPR43 and hydroxycarboxylic acid receptor 2 (HCAR2, GPR109A), participate in SCFAs-induced signaling cascades (27). These receptors are sensitive to pertussis toxin (PTX) and are linked to Gi/o-type G proteins. They are involved in the inhibition of adenylate cyclase and the promotion of AMP-dependent mechanisms, while having relatively limited influence on the phospholipase C (PLC) pathway. In addition, GPR43 interacts with Gq protein signaling through the PLC pathway (25). GPCRs can bind a wide range of extracellular substances, including hormones, carbohydrates, odor molecules, lipids, neurotransmitters, proteins and chemokines (28). After ligand activation, GPCRs interact with various heterotrimeric G proteins, including Gs, Gi/o, Gq/11 and G12/13. These heterotrimeric G proteins regulate the activity of one or more effectors, such as enzymes or ion channels that generate second messenger molecules (28). Studies have demonstrated that GPR41 and GPR43 receptors play important roles in the uptake of SCFAs and the affinity of different SCFAs for these receptors varies. According to previous studies, the affinity of GPR43 for SCFAs follows the order acetate>propionate>butyrate, while the affinity of GPR41 for SCFAs follows the order acetate>butyrate (29). Because of their involvement in mediating these conditions, GPR41 and GPR43 have become potential therapeutic targets for the treatment of metabolic disorders including T2DM (28). In summary, microbial-derived SCFAs play an important role in multiple physiological processes. Understanding the functions of microbial-derived SCFAs, their receptors and downstream signaling pathways provides valuable insights into the development of new therapeutic strategies for various health conditions.

GPR41-mediated inflammatory regulatory pathways mainly depend on the Mitogen-Activated Protein Kinase (MAPK) signaling axis. After propionate and butyrate are recognized by this receptor, cAMP production is inhibited through coupling with Gi/o protein, and Extracellular Signal-Regulated Kinase 1/2 (ERK1/2) and p38 Mitogen-Activated Protein Kinase (p38 MAPK) are subsequently activated in epithelial cells (30, 31). Activation of this pathway produces a bidirectional regulatory effect: on one hand, it directly down-regulates the expression of pro-inflammatory cytokines (IL-1, TNF-α) and several chemokines (CCL20, CXCL2/3/5/8/14); on the other hand, it up-regulates tight junction proteins Occludin and Zonula Occludens-1 (ZO-1), thereby reducing inflammatory cell infiltration and strengthening intestinal barrier integrity (32). In addition, GPR41 signaling also establishes an intestinal immune protective barrier by promoting IL-22 production from CD4 + T cells and congenital lymphocytes (33). GPR43 integrates multiple anti-inflammatory signaling pathways. This receptor performs several regulatory roles in immune cells such as adipose tissue cells, intestinal endocrine cells and neutrophils through Gi/o or Gq protein coupling (34, 35). At the level of signal transduction, GPR43 activation can suppress hyperglycemia-induced oxidative stress and NF-κB activation. The mechanism involves enhancing the interaction between β-arrestin2 and I-κBα, thereby blocking p65 nuclear translocation and downstream transcription of pro-inflammatory genes (36). At the same time, GPR43 promotes the production of antimicrobial peptides such as Reg3γ and β-defensins by intestinal endocrine cells through the mTOR/STAT3 pathway, maintains intestinal microecological homeostasis and indirectly suppresses inflammation initiation (37). In metabolic organs, GPR43 signaling reduces fat accumulation by inhibiting insulin signaling in adipocytes and promotes lipid utilization in other tissues. This regulatory mechanism is closely associated with the reduction of inflammation in adipose tissue (38). GPR109A constitutes a negative regulatory pathway of NOD-like receptor family pyrin domain containing protein 3 (NLRP3) inflammasome. As a butyrate specific receptor, GPR109A transmits signals through Gi/o proteins in colonic epithelial cells and immune cells (39). Its anti-inflammatory effects are reflected at three levels. First, it directly inhibits the activation of NLRP3 inflammasome and reduces caspase-1 dependent IL-1β maturation and secretion (40). Secondly, Foxp3 + T cells and IL-10 + T cells are indirectly induced to differentiate by acting on macrophages and dendritic cells (39, 41). In addition, activation of GPR109A in adipose tissue can improve glucose metabolism and suppress inflammatory responses (42).

### 2.2 HDAC inhibition and related pathways

The epigenetic regulatory function of SCFAs mainly relies on the inhibition of histone deacetylase (HDAC), which constitutes a central signaling pathway through which SCFAs regulate immune cell function, inflammatory responses and metabolic homeostasis. Histone acetylation is regulated by histone acetyltransferase (HAT) and HDAC, which catalyze the removal of acetyl groups, resulting in chromatin compaction and suppression of gene transcription (43). The human HDAC family contains 18 members, which are divided into four categories: class I (HDAC 1,2,3,8), class IIa (HDAC 4,5,7,9), class IIb (HDAC 6,10), class III (SIRT1-7) and class IV (HDAC 11). These enzymes are widely involved in the transcriptional regulation of inflammation, glucose metabolism and insulin signal transduction (44). Among all SCFAs, butyrate is the most active HDAC inhibitor, which systemically regulates immune function and whole-body metabolism through this pathway. The HDAC inhibition pathway mediates multiple anti-inflammatory signaling cascades. Butyrate enhances the antibacterial activity of macrophages by inhibiting HDAC, while simultaneously reducing the expression of nitric oxide (NO), thereby modulating the intensity of inflammation (45). During macrophage polarization, HDAC inhibition promotes the transformation toward the M2 anti-inflammatory phenotype and suppresses NLRP3 inflammasome activation. This effect involves transcriptional reprogramming caused by HDAC inhibition, enabling intestinal macrophages to develop tolerance to microbial stimulation (46). In neutrophils, propionate and butyrate enhance apoptosis by inhibiting HDAC activity, and this effect is independent of GPCR and MAPK signaling pathways, indicating that HDAC inhibition can regulate the survival of inflammatory cells as a parallel pathway independent of receptor-mediated signaling (47). In addition, butyrate can synergistically inhibit HDAC through GPR41/43 receptors, preventing bone marrow stem cells from differentiating into dendritic cells and thereby inhibiting the initiation of inflammatory responses at the level of immune cell differentiation (48). Arpaia et al. first demonstrated that butyrate produced by gut microbiota enhances histone acetylation levels in the Foxp3 gene promoter and conserved non-coding region by inhibiting HDACs—a critical epigenetic event in the establishment of Tregs lineage, which induces colonic Tregs differentiation by promoting Foxp3 expression and thereby maintains immune homeostasis (49). This mechanism illustrates how HDAC inhibition establishes a long-term immune tolerance state through chromatin remodeling. butyrate stabilizes the degradation of hypoxia-inducible factor (HIF), a transcription factor in intestinal epithelial cells under normoxic conditions, through HDAC inhibition; meanwhile, HDAC inhibition can delay its degradation process, thereby promoting the expression of barrier-protective genes and enhancing tight junction and barrier integrity (50). At the same time, butyrate can directly promote the assembly of tight junction proteins Occludin and ZO-1 by activating the AMPK pathway, which is also associated with HDAC inhibition and constitutes another parallel pathway that strengthens the epithelial barrier (51). These two pathways HIF stabilization and AMPK activation—together form a barrier protection signaling network downstream of HDAC inhibition. In the streptozotocin-induced diabetic rat model, sodium butyrate treatment increased plasma insulin levels, decreased blood glucose levels, and reduced pancreatic β-cell apoptosis by inhibiting HDAC to regulate p38/ERK and apoptosis pathways (52). This observation indicates that HDAC inhibition not only influences immune cells but also directly affects cell fate determination signaling pathways in metabolic organs.

## 3 Function of SCFAs on T2DM-related immune regulation

**Figure 4:**
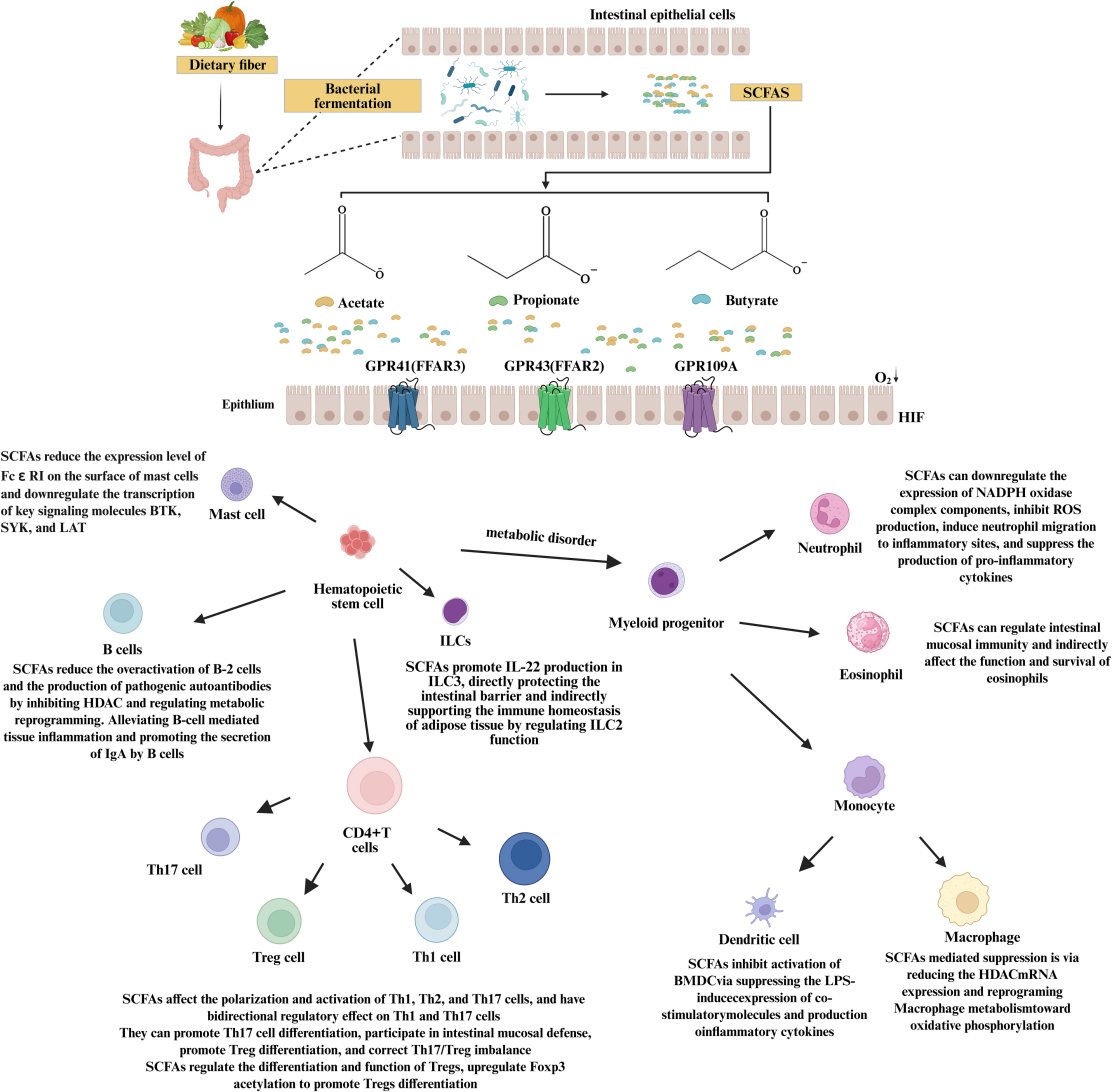
The network of T2DM-related immune cells regulated by SCFAs.

SCFAs play an important role in regulating the immune system and inflammatory responses. SCFAs exert key regulatory effects on the immune system, influencing both innate immune responses and adaptive immune responses. The “participants” in the innate immune system are located close to the intestinal cavity, whereas the adaptive immune components are influenced by the absorption of lower concentrations of SCFAs into the systemic circulation (Figure 4).

### 3.1 Innate immune cells

As the first line of defense against pathogens, innate immune cells play a crucial role in the initiation and amplification of inflammation during the development of T2DM. The coordinated activation of these innate immune cells not only constitutes the cellular basis of chronic low-grade inflammation in T2DM, but more importantly, they form a self-amplifying positive feedback loop through the release of inflammatory mediators and chemokines. Inflammatory responses recruit additional immune cells, and these recruited cells subsequently release more inflammatory mediators, allowing local inflammation to persist and progressively spread throughout the body. Ultimately, this process creates a relatively stable microenvironment that supports the occurrence and maintenance of insulin resistance.

#### 3.1.1 Macrophages

Macrophages are recognized to play a crucial role in the pathological processes of obesity or IR. There are two major subtypes of macrophages in adipose tissue. One subtype is pro-inflammatory macrophages, referred to as “M1,” which are cultured in the presence of granulocyte-macrophage colony-stimulating factor (GM-CSF); the other subtype is anti-inflammatory macrophages, referred to as “M2,” cultured in the presence of IL-4. M1 macrophages exhibit pro-inflammatory activity by secreting high levels of pro-inflammatory markers and cytokines, whereas M2 macrophages show increased secretion of anti-inflammatory cytokines (such as IL-4 and IL-10) (53). Macrophages constitute the first line of defense and play a protective role in immune responses, and their functions can be modified by SCFAs (54). However, they can also respond appropriately to the microbiota to regulate microbial populations without inducing inflammation (55). During disease development, the metabolic profile of activated macrophages may change. Studies have shown that M1 macrophages shift their metabolism toward the glycolysis pathway. The gut microbiota exerts significant influence on various metabolic pathways, mainly through the production of SCFAs. Butyrate can alter metabolic pathways in macrophages, leading to increased oxidative phosphorylation (OXPHOS) and fatty acid metabolism, ultimately promoting the M2 phenotype (56). The resident macrophage population represents an important defense mechanism against pathogens in the digestive tract (57). However, it should be noted that circulating blood monocytes are the main source of intestinal macrophages. Macrophages in the gut represent a phenomenon that contrasts markedly with observations in many other organs of the body (58). Final differentiation occurs in the lamina propria, which is located close to symbiotic bacteria. Schulthess et al. (59) conducted experiments to investigate the potential effects of SCFAs macrophages. Metabolic analysis of butyrate-treated macrophages showed that glycolysis was significantly reduced. It has been demonstrated that butyrate can inhibit the activation of NF-κB in macrophages. NF-κB is a eukaryotic transcription factor involved in the regulation of multiple cellular processes, including immune and inflammatory responses (60). Butyrate, propionate and acetate have been shown to influence the production of pro-inflammatory cytokines and inhibit the activity of inducible nitric oxide synthase in macrophages, thereby suppressing inflammatory responses (61). SCFAs have been reported to inhibit the inflammatory-activated macrophage phenotype and promote its conversion into an anti-inflammatory M2-polarized macrophage phenotype, as observed in dextran sulfate sodium colitis and alcoholic hepatitis mouse models (62).

#### 3.1.2 Neutrophils

Neutrophils play an important role in chronic low-grade inflammation associated with T2DM. Under conditions of obesity and IR, the number of circulating neutrophils increases significantly. After activation, these cells release elastase, myeloperoxidase and large amounts of reactive oxygen species (ROS), which not only directly cause tissue damage but also promote adipose tissue inflammation and macrophage activation through the formation of neutrophil extracellular traps (NETs), thereby further aggravating IR (63). SCFAs can influence neutrophil chemotaxis and survival induced by inflammatory mediators and chemokines. SCFAs can also regulate the chemotactic behavior of neutrophils. Studies have shown that butyrate treatment can lead to the accumulation of immature neutrophils in bone marrow and affect neutrophil mobilization to inflammatory sites by regulating C-X-C Motif Chemokine Receptor 2 (CXCR2) signaling (64). SCFAs can markedly inhibit the oxidative burst of neutrophils and reduce the production of ROS. Studies have shown that butyrate can down-regulate the expression of components of the NADPH oxidase complex. acetate, propionate and butyrate can significantly inhibit ROS production induced by bacterial stimulation (65). SCFAs-mediated activation of GPR43 can induce neutrophil migration to inflammatory sites and enhance their phagocytic capacity (66). However, SCFAs can also suppress the production of pro-inflammatory cytokines such as TNF-α in neutrophils (67). Overall, SCFAs can effectively restrain excessive neutrophil-mediated inflammatory responses, thereby playing a beneficial role in reducing T2DM-related tissue injury and improving IR.

#### 3.1.3 Dendritic cells (DCs)

As key antigen-presenting cells linking innate immunity and adaptive immunity, DCs play an important role in the pathological progression of T2DM. Under conditions of obesity and insulin resistance, the infiltration of DCs in adipose tissue and pancreatic islets increases. These DCs display a pro-inflammatory phenotype, activate CD4 + T cells through efficient antigen presentation and promote their differentiation into pro-inflammatory subsets such as Th1/Th17, thereby aggravating local tissue inflammation and IR (68). SCFAs (especially butyrate) influence the differentiation of monocytes into DCs by inhibiting HDAC activity. Studies have shown that butyrate treatment can suppress the acquisition of Cluster of Differentiation 1a (CD1a) and up-regulate the expression of CD1d during the differentiation of monocyte-derived DCs, suggesting that SCFAs may regulate DC lineage differentiation through epigenetic mechanisms (69). SCFAs can also induce DCs to acquire a tolerogenic phenotype. butyrate can induce the expression of retinal dehydrogenase 1 (RALDH1) in human monocyte-derived DCs by simultaneously inhibiting HDAC and activating G protein-coupled receptor GPR109 A signaling. This enzyme catalyzes the production of retinoic acid, which subsequently promotes the differentiation of naive CD4 + T cells into IL-10-producing type 1 regulatory T cells (Tr1) (70). SCFAs butyrate and propionate inhibit the activation of bone marrow-derived dendritic cells (BMDCs) by suppressing the expression of lipopolysaccharide (LPS)-mediated costimulatory molecules (such as CD40) and the production of cytokines (such as IL-6 and IL-12p40) (39). In summary, SCFAs may reshape the immunomodulatory functions of DCs, thereby exerting potential therapeutic value in reducing T2DM-related tissue inflammation and improving IR.

#### 3.1.4 Mast cells

Mast cells play a complex and critical role in the pathological process of T2DM. In conditions of obesity and IR, mast cells accumulate and become activated in adipose tissue, releasing histamine, proteases and various pro-inflammatory mediators. These mediators promote macrophage recruitment, adipose tissue fibrosis and inflammatory responses, thereby aggravating local tissue IR and systemic metabolic disorders (71). It is noteworthy that recent studies have revealed dynamic changes in mast cell phenotype in adipose tissue of patients with T2DM. In the omental adipose tissue of patients with mild T2DM, the expression of Cluster of Differentiation 45 (CD45), CD117, CD203c and High-Affinity Immunoglobulin E Receptor (FcεRI) on the surface of mast cells was significantly decreased, which may reflect adaptive regulation under chronic metabolic stress conditions (72). SCFAs inhibit IgE-mediated mast cell degranulation by activating the GPR109A receptor, a process that depends on the Gi protein signaling pathway and the downstream release of prostaglandin E2 (PGE2). PGE2 subsequently suppresses mast cell activation by acting on the EP3 receptor (73). SCFAs exert epigenetic regulation by inhibiting HDAC activity. Butyrate treatment can significantly reduce the expression level of FcεRI on the surface of mast cells and down-regulate the transcription of key signaling molecules Bruton’s Tyrosine Kinase (BTK), Spleen Tyrosine Kinase (SYK) and Linker for Activation of T Cells (LAT), which represent core kinases required for FcεRI-mediated mast cell activation (74). In addition, transcriptomic analysis has shown that butyrate induced HDAC inhibition can alter the global histone acetylation pattern in mast cells, particularly reducing acetylation levels in the promoter regions of BTK, SYK and LAT, thereby persistently inhibiting mast cell activation potential at the epigenetic level (74). Through the dual mechanisms of GPR109A receptor signaling and HDAC inhibition-mediated epigenetic regulation, SCFAs can effectively suppress excessive mast cell activation and the release of inflammatory mediators, thereby showing potential therapeutic value in alleviating T2DM-related adipose tissue inflammation and improving IR.

#### 3.1.5 Eosinophils

Eosinophils play a distinctive protective role in maintaining adipose tissue immune homeostasis associated with T2DM. In healthy adipose tissue, eosinophils maintain local immune balance by secreting cytokines (such as IL-4 and IL-13). These cytokines promote macrophage polarization toward the M2 anti-inflammatory phenotype and support the maintenance of Tregs, thereby forming an immune regulatory network that maintains insulin sensitivity in adipose tissue (75). Studies have shown that eosinophils represent the main source of IL-4 in white adipose tissue. In eosinophil-deficient mice, M2 macrophages in adipose tissue were significantly reduced, and mice subjected to high-fat diet (HFD) induction exhibited more severe glucose intolerance and IR (76). Under conditions of obesity, the number of eosinophils in adipose tissue decreases, leading to weakened immune regulation and the conversion of M2 macrophages to the M1 pro-inflammatory phenotype, thereby aggravating local inflammation and metabolic disorders (77). The regulatory mechanism of SCFAs on eosinophils has not been fully elucidated, but there is evidence that SCFAs may indirectly influence the function and survival of eosinophils by regulating intestinal mucosal immunity (78). Based on the known roles of SCFAs in regulating ILC2 function and macrophage polarization, it is speculated that SCFAs may affect eosinophil recruitment, survival and cytokine secretion in adipose tissue through GPR41/43 receptor signaling or HDAC inhibition. Through these mechanisms, SCFAs may indirectly support M2 macrophage polarization and help maintain adipose tissue immune homeostasis. This area requires further investigation.

#### 3.1.6 ILCs

ILCs are key effector cells of innate immunity and can produce regulatory and pro-inflammatory cytokines that promote tissue repair, immune responses and inflammation (79). Mature ILCs lack T cell receptors (TCRs). According to their surface markers, cytokine production and transcription factor expression, ILCs can be classified into type 1, type 2 and type 3 (80). These subsets correspond to different types of CD4 + helper T cells, including Th1, Th2 and Th17. ILC1 is characterized by the production of IFN-γ, whereas cytokines such as IL-5 and IL-13 are mainly produced by ILC2, and the primary products of ILC3 are IL-17 and IL-22. ILCs play an important role in maintaining immune homeostasis in intestinal and adipose tissues associated with T2DM. In conditions of obesity and metabolic disorders, ILC subsets exhibit significant imbalance. The number of pro-inflammatory ILC1 (similar to Th1, secreting IFN-γ) increases, whereas the number of tissue-protective ILC2 and ILC3 decreases. This imbalance directly influences intestinal barrier integrity and immune homeostasis in adipose tissue (81). SCFAs exert differential regulatory effects on ILC subsets. At the level of ILC3 regulation, recent studies have shown that dietary fiber-derived acetic acid directly acts on ILC3 through its receptor FFAR2 and promotes IL-22 production. IL-22 is a key cytokine for maintaining intestinal barrier function. It can inhibit excessive expression of MHC-II in colonic epithelial cells, thereby preventing the expansion of pathogenic CD4^+^ intraepithelial lymphocytes and protecting the intestine from inflammatory damage (82). This mechanism highlights the central role of the SCFAs-ILC3-IL-22 axis in maintaining intestinal immune homeostasis. At the level of ILC2 regulation, although the direct regulation of ILC2 by SCFAs has not been fully elucidated, current evidence suggests that ILC2 indirectly supports eosinophil survival and M2 macrophage polarization by secreting cytokines such as IL-5 and IL-13 (81). In healthy adipose tissue, the type 2 immune response mediated by ILC2 contributes to maintaining the homeostasis of eosinophils and M2 macrophages, thereby supporting insulin sensitivity in adipose tissue. Therefore, SCFAs may directly protect intestinal barrier function by promoting IL-22 production from ILC3, while indirectly supporting adipose tissue immune homeostasis through the regulation of ILC2 function. These coordinated processes together represent an important mechanism through which SCFAs improve T2DM-related metabolic disorders. Future studies should further clarify the direct regulatory mechanisms of SCFAs on ILC2 and their specific roles in adipose tissue immune metabolism.

### 3.2 Adaptive immune cells

Adaptive immune cells, with their antigen specificity and immune memory characteristics, play an important role in maintaining chronic inflammation in T2DM. On the basis of innate immune activation, these adaptive immune cell alterations allow local inflammation to persist and gradually expand into a systemic state, forming a self-sustaining chronic low-grade inflammatory cycle. Different from the rapid response of innate immunity, the participation of adaptive immunity gives T2DM immune disorders more persistent and systemic characteristics, which also represents an important basis for the chronic progression of the disease.

#### 3.2.1 CD4 + T cells

CD4 + T cells play an important role in the pathological process of obesity and IR. According to their function and the types of cytokines they produce, CD4 + effector T cells can be further subdivided into pro-inflammatory Th1 and Th17 subsets and anti-inflammatory Th2 and Foxp3 + T cell subtypes. The balance between Th2 or Treg and Th1 or Th17 effector T cell subsets is critical for maintaining immune homeostasis and proper immune responses. A large body of evidence indicates that CD4 + T cell differentiation is imbalanced in obese patients with T2DM (83). Th1 cells are differentiated CD4 + T cells characterized by the production of pro-inflammatory IFN-γ. Th2 cells are derived from activated CD4 + T cells and promote the production of lineage-defining cytokines such as IL-4, IL-5 and IL-13. When CD4 + T cells encounter IL-6 and transforming growth factor-β (TGF-β), they frequently differentiate into Th17 cells, which play pathogenic roles in many inflammatory diseases (84). SCFAs can induce the differentiation of CD4 + CD25 + Foxp3 + T cells in human peripheral blood mononuclear cells and increase the secretion of the anti-inflammatory cytokine IL-10 (85). Butyrate has been shown to promote the polarization of peripheral cells outside the thymus into Tregs cells both *in vitro* and *in vivo* (49). SCFAs can also influence the polarization and activation of Th1, Th2 and Th17 cells. *In vitro* experiments have demonstrated that acetate, propionate and butyrate can promote the production of Th1 and Th17 cells (86). Butyrate exhibits a concentration-dependent bidirectional regulatory effect on Th1 and Th17 cells. At lower concentrations, it promotes Th17 cell differentiation and participates in intestinal mucosal defense. At higher concentrations, butyrate markedly inhibits Th17 cell differentiation and promotes Tregs differentiation by activating the PPARγ pathway and regulating energy metabolism reprogramming, thereby correcting the Th17/Tregs imbalance (87).

#### 3.2.2 CD8 + T cells

CD8 + T cells are essential for adaptive immune responses to infection through the secretion of cytokines such as IFN-γ and TNF-α. Previous studies have also confirmed that CD8 + T cells can synthesize and express the pro-inflammatory cytokine IL-17, which is present in inflammatory tissues in many human inflammatory diseases (88). Earlier studies have shown that the accumulation of these cells can induce inflammation and IR. Patients with T2DM or mice fed a HFD appear to have a higher proportion of CD8 + T cells (89). Studies have demonstrated that SCFAs regulate the growth, differentiation, metabolism, effector function and apoptosis of CD8 + T cells through HDAC inhibition, GPCR signaling and metabolic reprogramming (90). Butyrate treatment significantly increased the frequency of IFN-γ-producing effector cells in activated CD8 + T cells and enhanced cytokine production in individual cells. It also up-regulated TCR expression and mTOR protein phosphorylation, thereby promoting the formation of an effector memory phenotype (91). Butyrate and propionic acid regulate CD8^+^T cell activation by inhibiting IL-12 production in DCs. However, microbiota-derived SCFAs can also enhance the function of CD8 + T cells by altering cellular metabolism (92). Acetate can further promote IFN-γ production in CD8 + T cells by regulating mTOR activity and cellular metabolic pathways.

#### 3.2.3 Tregs

Tregs express CD4, CD25 and Foxp3. They represent a small subset of T lymphocytes, accounting for only 5 to 20% of CD4 + cells. These cells suppress effector T cell responses, limit inflammatory reactions and prevent autoimmunity (93). In patients with T2DM, the balance between Tregs and Th1 or Th17 cells is crucial for maintaining proper immune responses. Treg cells can improve insulin resistance by inhibiting the activity of Th1, Th2 and Th17 cells. It has been reported that the proportion of Tregs cells in the peripheral blood of patients with T2DM is reduced, particularly in newly diagnosed patients, which contributes to the progression of inflammation and IR (94). The differentiation and function of Tregs can be regulated by metabolites derived from gut microbiota, including SCFAs. Tregs express the transcription factor Foxp3 and differentiate either in the thymus or in peripheral tissues. These cells represent an important regulatory component in immune tolerance and immune modulation. SCFAs can regulate T cell function through GPCR signaling, which is essential for maintaining intestinal epithelial physiology and plays a direct role in inducing Tregs in the intestine (33). They can promote the transformation of naive T cells into Tregs (95). Since SCFAs can enter the systemic circulation, they may exert broader systemic effects. Indeed, an increase in Foxp3 + Tregs has been observed in mice treated with SCFAs (96). Butyrate can also up-regulate histone H3 acetylation at the Foxp3 locus, thereby promoting Tregs differentiation (96). In addition, SCFAs such as butyric acid can regulate DCs in both mice and humans to promote Tregs differentiation. After butyric acid exposure, DCs promote the differentiation of Foxp3 + Tregs and inhibit IFN-γ-producing cells through indoleamine 2,3-dioxygenase 1 (IDO1) and aldehyde dehydrogenase 1A2 (Aldh1A2) (97). Notably, SCFAs also promote the production of IL-10 in Th1, Th17 and Tregs cells (86).

#### 3.2.4 B cells

B cells have been shown to play a central role in the development of IR by producing IgG antibodies and activating T cells and macrophages (98). van Beek et al. showed that the expression of the activation marker CD38 on circulating B cells was significantly higher in obese patients with normal glucose tolerance than in obese patients with T2DM. B cells play an important role in responses to microbial infections and pathogen clearance. These cells not only produce antibodies but also release a variety of cytokines (99). Activation of vitamin D receptor (VDR) mediated by bile acid metabolites can reduce the continuous proliferation of B lymphocytes and induce apoptosis of activated B cells (100), while also inhibiting the production of immunoglobulin (Ig) (101). However, SCFAs can also stimulate glycolysis in B cells by activating mTOR. Acetyl-CoA produced by SCFAs is essential for plasma cell differentiation and antibody production (102). SCFAs can further promote the secretion of immunoglobulin A (IgA) by B cells (103). By inhibiting HDAC activity and regulating metabolic reprogramming, SCFAs can reduce excessive activation of B-2 cells and the production of pathogenic autoantibodies, thereby alleviating B cell-mediated tissue inflammation (104).

## 4 Discussion

The study of SCFAs as core metabolites of intestinal flora in the immune regulation of T2DM provides a new perspective for understanding the nature of metabolic diseases. Based on current evidence, several broader insights can be drawn.

SCFAs reveal the deeper biological logic underlying the co-evolution of host and microbiota. Mammals and gut microbes have formed a complex reciprocal relationship during millions of years of co-evolution. As chemical messengers in this relationship, SCFAs transmit an “environmental report” from the microbiota to the host—reflecting whether dietary fiber intake is sufficient, whether microbial composition remains balanced, and whether the intestinal environment is stable. The immune system senses these signals through receptors such as GPR41/43/109A and converts them into appropriate immune responses. When this ancient communication mechanism is disrupted by modern lifestyles—reduced dietary fiber intake, loss of microbial diversity and decreased SCFAs production—the immune system loses important regulatory signals and shifts toward a persistent pro-inflammatory state, eventually contributing to “civilization-associated diseases” such as T2DM. In this sense, research on SCFAs not only reveals specific molecular mechanisms but also encourages reconsideration of the position of metabolic diseases within the broader context of human evolutionary history.

The “dual-track regulation” model of SCFAs reflects the intricate design of biological systems. Receptor-mediated rapid signal transduction and HDAC inhibition-mediated long-term epigenetic programming represent two temporal dimensions through which immune cells process environmental information. The former allows cells to respond quickly to short-term fluctuations, whereas the latter provides a form of cellular memory of long-term environmental trends. This regulatory architecture enables the immune system to respond flexibly to rapidly changing environmental conditions while maintaining long-term homeostasis, achieving a delicate balance between rapid adaptation and sustained stability. The development of T2DM may result from disruption of this balance. Long-term SCFAs deficiency may shift epigenetic programming in immune cells, causing the immune system to remain persistently biased toward a pro-inflammatory state, and even short-term interventions may struggle to reverse this “pathological memory.” This observation suggests that prevention may be more fundamental than treatment, because rebuilding disrupted epigenetic homeostasis is far more difficult than temporarily suppressing inflammatory responses.

The complexity of SCFAs regulatory networks challenges the traditional “single-target” paradigm of drug development. Unlike highly selective synthetic drugs, SCFAs act simultaneously on multiple immune cell types, integrate various signaling pathways and influence several metabolic organs, restoring homeostasis at a systemic level rather than blocking a single molecular target. This multi-target characteristic may explain why dietary interventions have shown unique advantages in the prevention and management of chronic diseases. Metabolic diseases essentially represent systemic network imbalances rather than isolated molecular defects. This raises an important question: while pursuing “precision medicine,” has the complexity of disease mechanisms been oversimplified? It may be necessary to reconsider the value of traditional concepts of “multi-target, systemic regulation” within modern medicine.

The “environmental dependence” of SCFAs also challenges the simple dichotomy between “beneficial” and “harmful.” Low concentrations of SCFAs can promote Th17 cells to participate in intestinal defense, whereas higher concentrations inhibit Th17 differentiation and promote Treg expansion. SCFAs may induce anti-inflammatory phenotypes under steady-state conditions but enhance antibacterial immunity during infection. Activation may occur in one cell type while inhibition occurs in another. This context-dependent characteristic indicates that SCFAs are not merely “anti-inflammatory molecules,” but rather “tuners” of immune responses. Their biological effects depend on cell type, local concentration, microenvironmental signals and pathological conditions. This observation suggests that when translating experimental findings into clinical interventions, the linear assumption of “higher dose produces better outcomes” should be avoided. Instead, the complex parameter space defined by dosage, location, timing and individual variability should be carefully considered.

From a broader perspective, research on SCFAs is contributing to a shift in the medical paradigm from “treating disease” to “maintaining health.” By optimizing dietary structures to support SCFAs-producing microbiota, regulating microbial metabolism to train immune responses and restoring symbiotic relationships between host and microorganisms, intervention strategies based on ecological principles may offer more fundamental and sustainable benefits than drugs targeting a single pathway. Following this conceptual framework may allow medicine to move beyond the limitations of “fighting disease” and toward a broader future focused on “nurturing health.” In this sense, the study of SCFAs not only clarifies specific molecular mechanisms but also encourages reconsideration of the concept of health itself: health is not merely the absence of disease, but the result of harmonious communication between the host and the microbiota.

## 5 Limitations and future directions

Although significant progress has been achieved in studies investigating the role of SCFAs in the immune regulation of T2DM, several key issues in this field still require further clarification.

From the perspective of mechanism, the spatio-temporal synergy between receptor signaling and epigenetic regulation has not been fully elucidated. How these two pathways achieve functional integration in specific cell types, the distinct roles of different HDAC subtypes in immune regulation, and how SCFAs concentration gradients determine the direction of cellular responses remain insufficiently understood. Future studies should construct a more detailed regulatory network of SCFAs by applying advanced techniques such as single-cell multi-omics and spatial transcriptomics.

From the perspective of research methods, current investigations face several technical limitations. *In vitro* experiments are difficult to reproduce the complex concentration gradients and intercellular interactions present *in vivo*. In addition, differences in immune system characteristics and microbiota composition between animal models and humans restrict the extrapolation of experimental conclusions. The heterogeneity of microbiota and metabolism among individuals also makes the reproducibility of results more challenging. In the future, it will be necessary to develop organoid co-culture systems, humanized animal models and organ-on-chip technologies to construct research platforms that more closely reflect physiological conditions.

From the perspective of clinical translation, the direct use of SCFAs for the treatment of T2DM still faces multiple obstacles. Low oral bioavailability, difficulties in targeted delivery, substantial individual variation in response and uncertain long-term safety limit the clinical application of SCFAs-related interventions. Future translational research should focus on developing efficient delivery systems, establishing precise patient stratification strategies, exploring potential synergy with existing drugs and designing selective agonists targeting SCFAs receptors.

Looking forward, SCFAs research is gradually progressing from “phenomenon description” toward “mechanistic analysis” and is increasingly extending into “clinical translation.” Investigating the migration networks of immune cells along the gut-fat-liver axis, establishing disease classification systems based on microbiota and immune characteristics, and developing interventions capable of regulating endogenous SCFAs production will represent important directions for future research. Ultimately, these studies will contribute to a deeper understanding of the biological significance of host-microorganism symbiosis and may open new avenues for the prevention and treatment of metabolic diseases.
